# Supplementary material for: DirectContacts2: A network of direct physical protein interactions derived from high-throughput mass spectrometry experiments
Source: bioRxiv. 2025 Aug 12:2025.07.17.665435. Preprint. [Version 3] doi: 10.1101/2025.07.17.665435 (PMC12324205; doi:10.1101/2025.07.17.665435)

bioRxiv preprint doi: https://doi.org/10.1101/2025.07.17.665435; this version posted August 12, 2025. The copyright holder for this preprint (which was not certified by peer review) is the author/funder, who has granted bioRxiv a license to display the preprint in perpetuity. It is made available under aCC-BY 4.0 International license.

Supplemental Figures

Supplemental Figure 1: Proteomics dataset coverage of DirectContacts2. A. Coverage of each proteomic experimental evidence type for human protein pairs. **B.** Coverage of each proteomic experimental evidence type for human proteins.

Supplemental Figure 2: Evaluation of DirectContacts2 Network A. Distribution of DirectContacts2 scores for all predicted pairs with score > 0.2. The distribution shows a bimodal distribution highlighting confident (>0.7) and highly confident (>0.9) predicted protein pairs. **B**. Distribution of DirectContacts2 scores for true direct interactions (green) and false indirect interactions (gray) in the leave-out test set derived from PDB structures of protein complexes. **C.** Precision-recall curve generated using the test set with inter-complex negative protein pairs (i.e. from separate complexes). DirectContacts2 score thresholds ≥ 0.7 (confident) and ≥ 0.9 (highly confident) are marked. **D**. Comparison of enrichment for high-confidence pair interfaces out of the set of top confident pairs for DirectContacts2 and other networks. For each set, the fraction of high-confidence interfaces (pDockQ ≥ 0.23) out of their top predictions was calculated for increasing numbers of top predictions.

bioRxiv preprint doi: https://doi.org/10.1101/2025.07.17.665435; this version posted August 12, 2025. The copyright holder for this preprint (which was not certified by peer review) is the author/funder, who has granted bioRxiv a license to display the preprint in perpetuity. It is made available under aCC-BY 4.0 International license.

Supplemental Figure 3: Area under the precision recall curve for top performing features to discriminate between direct and indirect interactions. Bar plot shows the area under the precision recall curve for untrained individual features evaluated on a training set excluding intercomplex negative edges. CFMS features are shown in green, APMS features are shown in pink, and WMM features are shown in orange. The two high ranking WMM features (neg_ln_pval_youn_hygeo_gt4 and neg_ln_pval_cilium_hygeo) are from proximity labeling experiments. Three random shufflings are also evaluated (gray).

Supplemental Figure 4: Weighted Matrix Model applied to proximity labeling experiments provides evidence for direct interactions in CCT/Prefoldin complex. A. Heatmap of proximity labeling experiments which observe presence of prefoldin subunits. Directly interacting pairs (PFDN5-PFDN3 (yellow box) and PFDN3-PFDN2 (purple box)) are seen together in the same experiments. **B.** PDB structure of CCT/Prefoldin complex showing PFDN5-PFDN3 and PFDN3-PFDN2 directly interact. CCT subunits in gray, PFDN5 (green), PFDN3 (orange), PFDN2 (pink), PFDN1 (red), PFDN6 (blue). **C.** Distribution of WMM calculated -lg(pval) of prefoldin subunit pairs shows bimodal distribution. Directly interacting pairs PFDN3-PFDN2 and PFDN5-PFDN3 are right shifted with higher WMM values.

Supplemental Tables(csv files with headers or excel files**)**

Supplemental Table 1: Benchmark Training Positive Pairs
Supplemental Table 2: Benchmark Train Negative Pairs
Supplemental Table 3: Benchmark Test Positive Pairs
Supplemental Table 4: Benchmark Test Negative Pairs
Supplemental Table 5: Benchmark of Test Inter-Complex Negative Pairs
Supplemental Table 6: Benchmark Positive Pairs with PDBs
Supplemental Table 7: AutoGluon leaderboard of models
Supplemental Table 8: All 26 million predictions
Supplemental Table 9: Confident DirectContacts2 Predictions
Supplemental Table 10: AlphaFold3 model prediction metrics

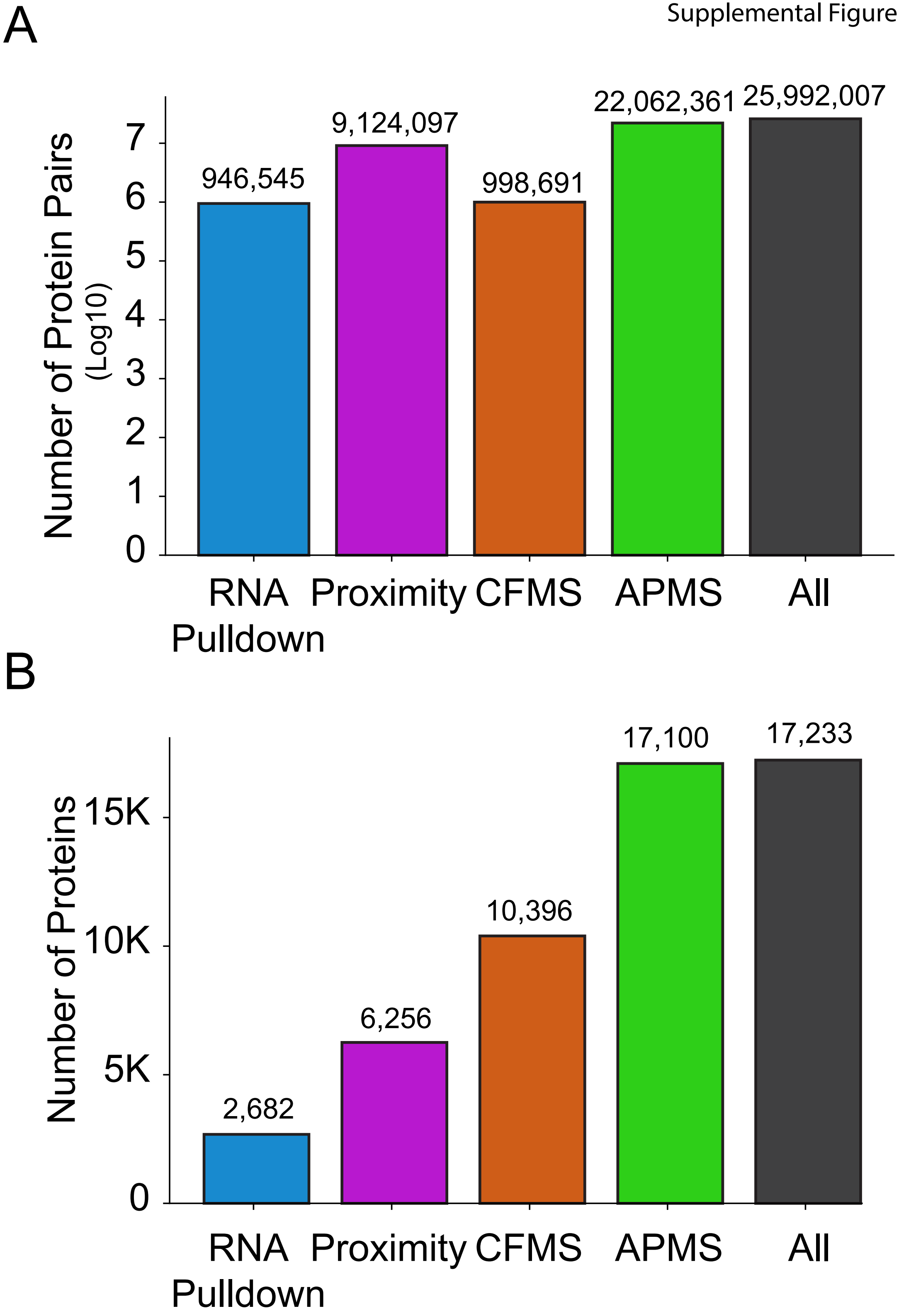
A
Number of Protein Pairs
(Log10)
0
1
2
3
4
5
6
7
946,545
9,124,097
998,691
22,062,361
25,992,007
RNA Pulldown
Proximity
CFMS
APMS
All
B
Number of Proteins
0
5K
10K
15K
2,682
6,256
10,396
17,100
17,233
RNA Pulldown
Proximity
CFMS
APMS
All

Supplemental Figure 2

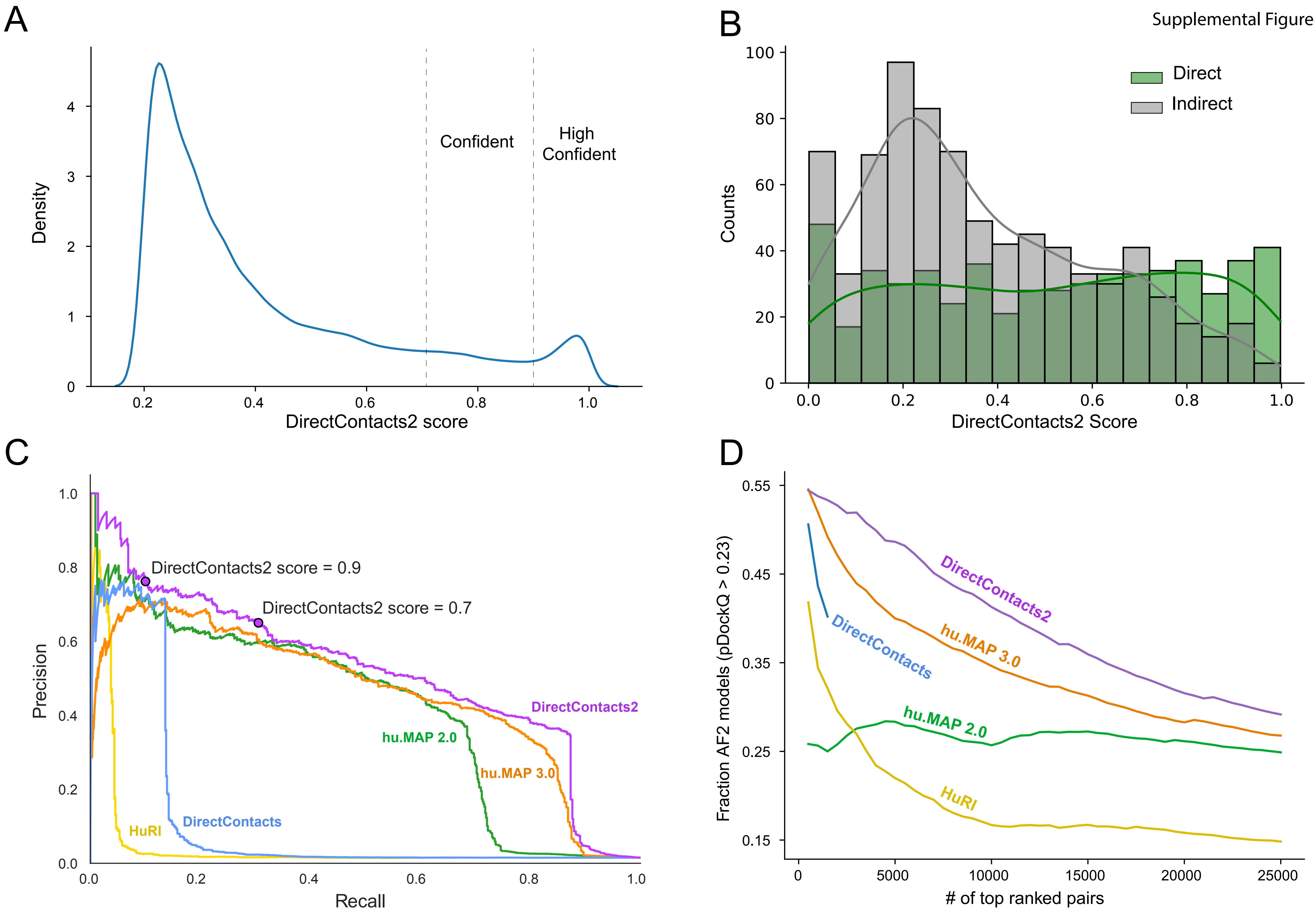

Supplemental Figure 3

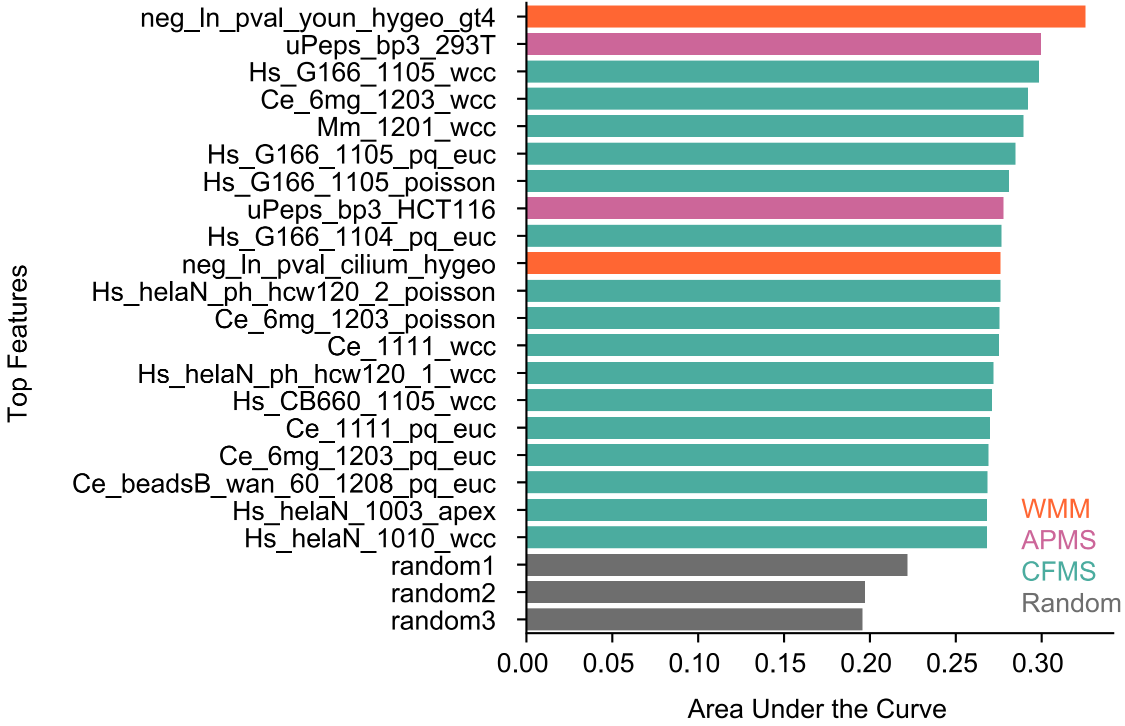

Supplemental Figure 4

A

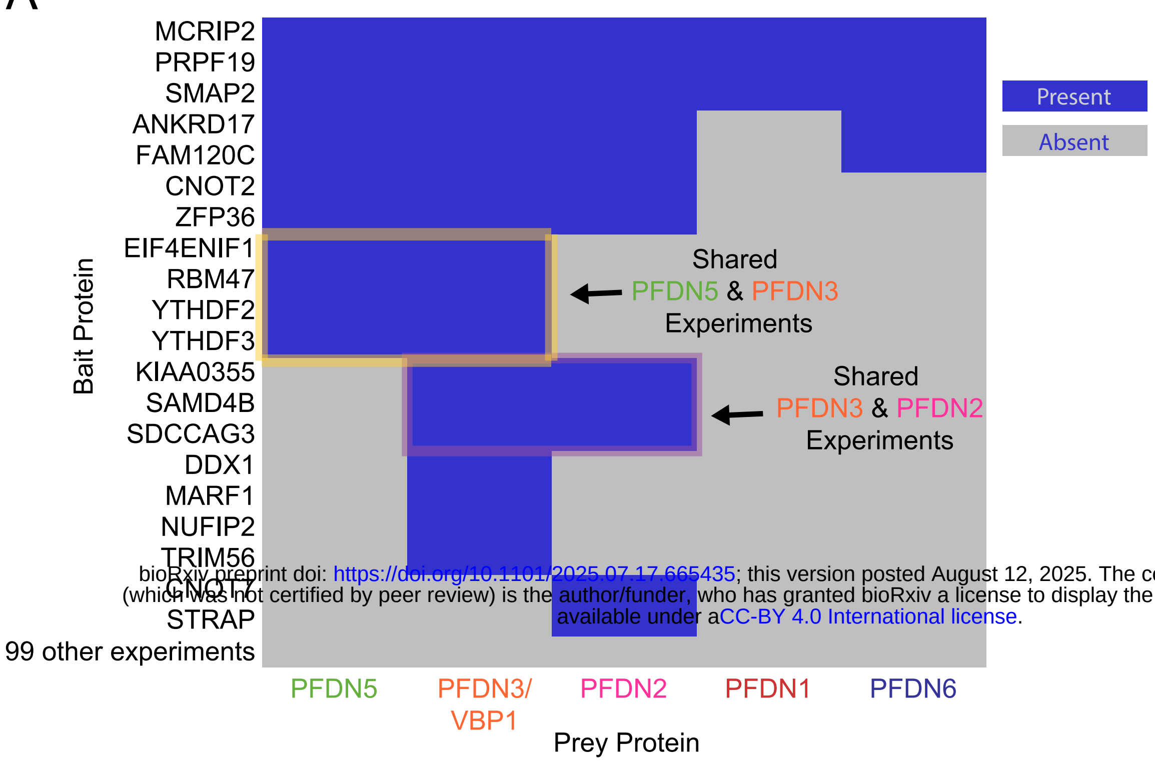

bioRxiv preprint doi: https://doi.org/10.1101/2025.07.17.665435; this version posted August 12, 2025. The copy (which was not certified by peer review) is the author/funder, who has granted bioRxiv a license to display the pre available under aCC-BY 4.0 International license.

B

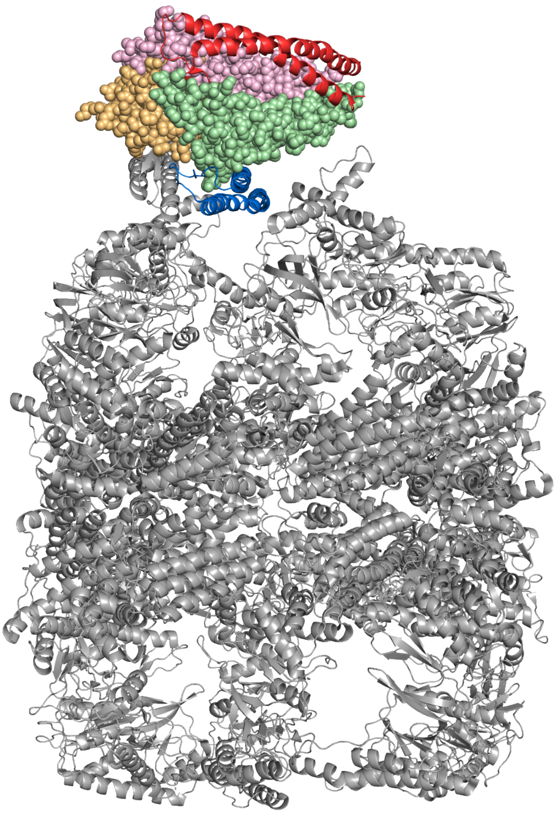

C

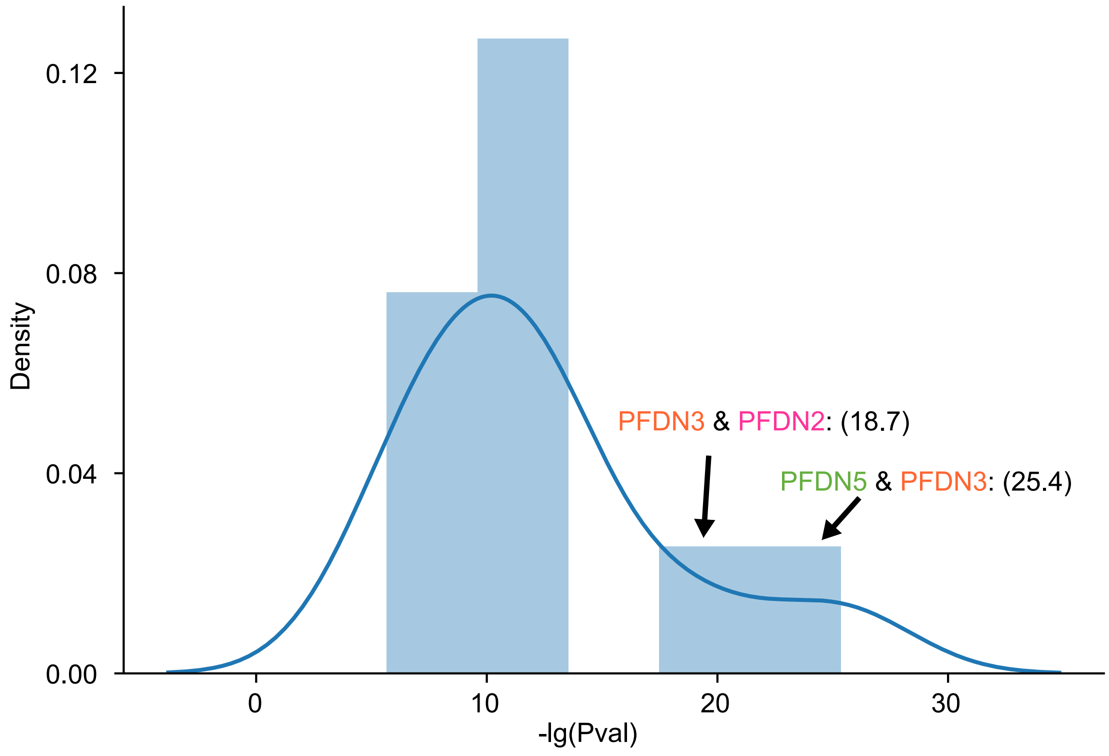

Supplement: 1 [file NIHPP2025.07.17.665435V3-supplement-1.pdf]
