# Supplementary material for: DirectContacts2: A network of direct physical protein interactions derived from high-throughput mass spectrometry experiments
Source: bioRxiv. 2025 Jul 28:2025.07.17.665435. Preprint. [Version 2] doi: 10.1101/2025.07.17.665435 (PMC12324205; doi:10.1101/2025.07.17.665435)
Supplement: Supplementary file 1 [file NIHPP2025.07.17.665435v2-supplement-11.pdf]

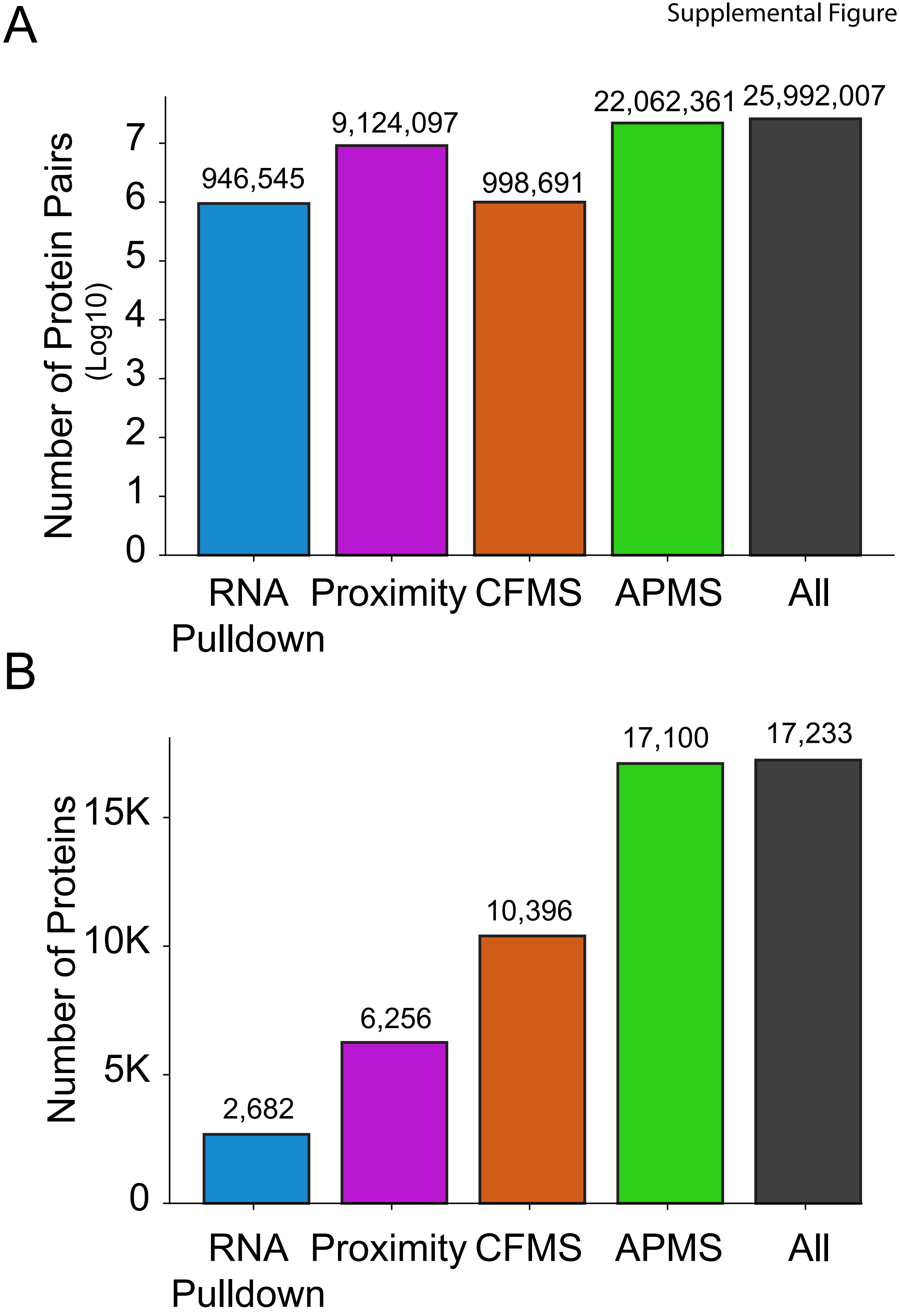
A
Number of Protein Pairs
(Log10)
0
1
2
3
4
5
6
7
946,545
9,124,097
998,691
22,062,361
25,992,007
RNA Pulldown
Proximity
CFMS
APMS
All
B
Number of Proteins
0
5K
10K
15K
2,682
6,256
10,396
17,100
17,233
RNA Pulldown
Proximity
CFMS
APMS
All

Supplemental Figure 2

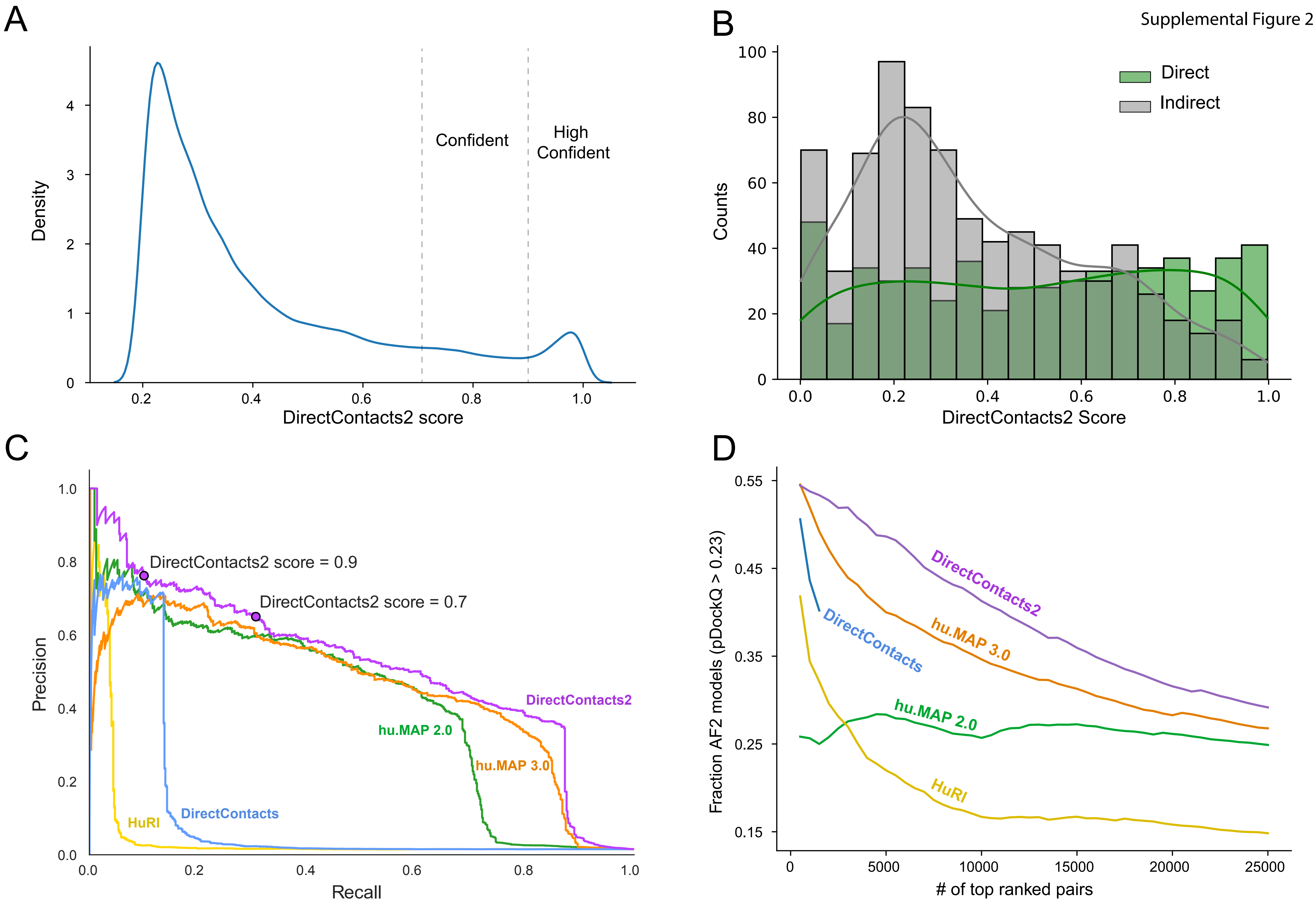

Supplemental Figure 3

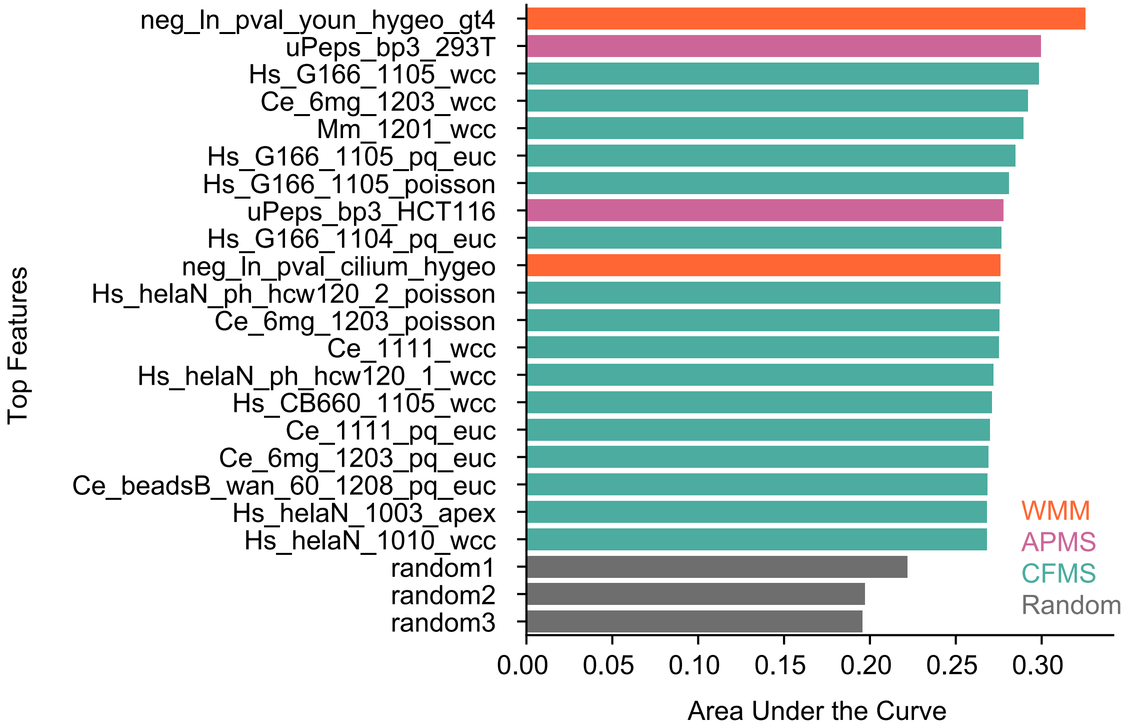

Supplemental Figure 4

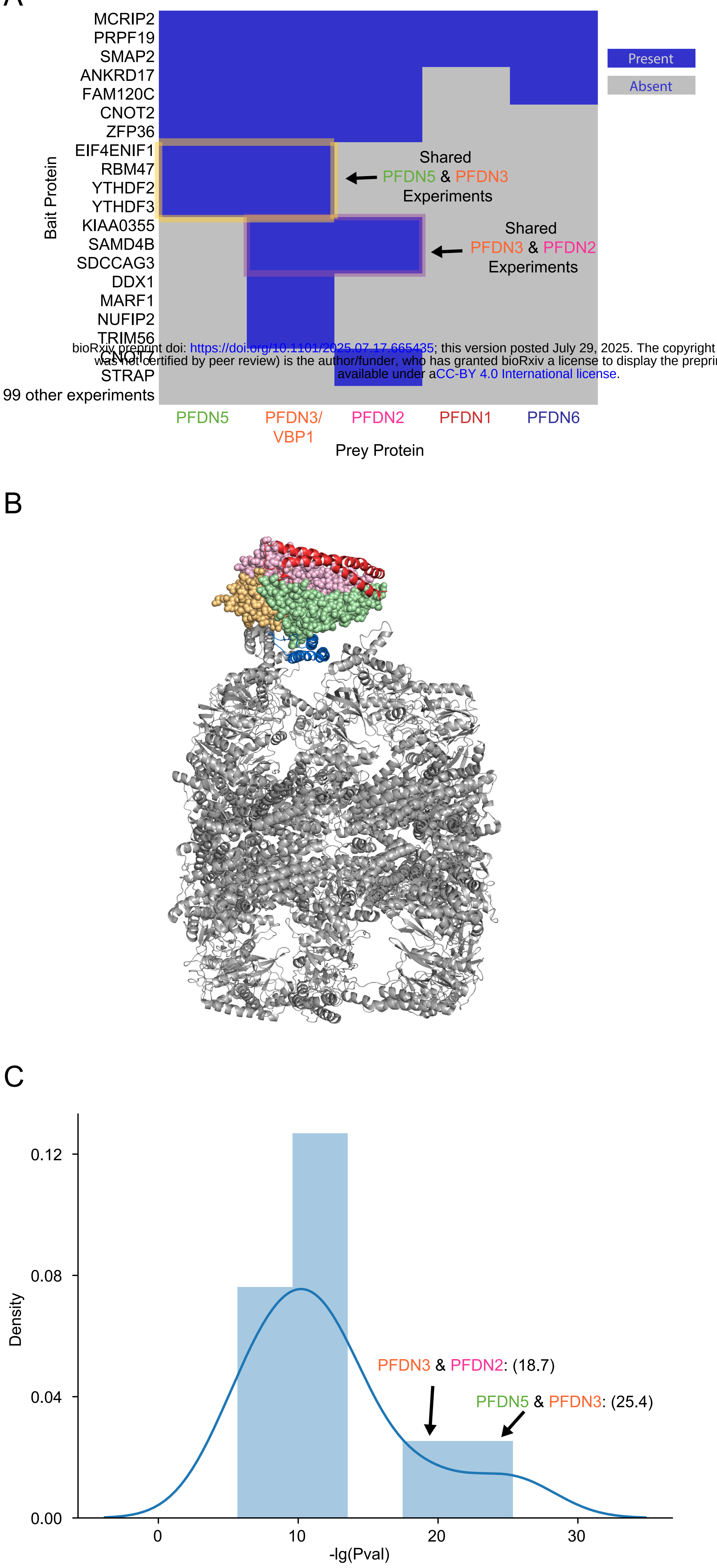

bioRxiv preprint doi: https://doi.org/10.1101/2025.07.17.665435; this version posted July 29, 2025. The copyright
was not certified by peer review) is the author/funder, who has granted bioRxiv a license to display the prepri
available under aCC-BY 4.0 International license.
